# Supplementary material for: The variations of native plasmids greatly affect the cell surface hydrophobicity of sphingomonads
Source: mSystems. 2023 Nov 1;8(6):e00862-23. doi: 10.1128/msystems.00862-23 (PMC10734547; doi:10.1128/msystems.00862-23)
Supplement: Supplemental figure legends — Legends for Fig. S1 to S6. [file msystems.00862-23-s0002.docx]

**Supplemental Figure Legends**

**FIG S1** Sequence alignments of the mutant proteins. The alignments were performed using Mega X with ClustalW alignment method.

**FIG S2** Sequence alignments of the p1 and p3 in the C1 and C2 genomes. **a** Sequence alignments of C1_p3 against C1_p1 and C2_p3. **b** Sequence alignments of C1 and C1n. The alignments were performed using Mauve. Each of colored blocks represents a presumably homologous region.

**FIG S3** The field emission scanning electron microscope images of C1, C2 and C2△*p3_rep*.

**FIG S4** The cell sizes of C1, C2 and their mutants. **a** The transmission electron microscope images of different strains. **b** The statistical analysis of cell sizes of different strains. Significant differences were tested by one-way ANOVA with LSD test (n > 30, α = 0.05). The a and b represent the differences among these strains.

**FIG S5** Hierarchical clustering of the transcriptomic profiles of C1, C2, C2n and C2△*p3_rep*. After removing batch effects, the overall gene transcriptional levels of C1, C2, C2n and C2△*p3_rep* were used to clustered.

**FIG S6** Genetic organization of the operon *wza_rfbCBDA*.
