# Supplementary material for: The variations of native plasmids greatly affect the cell surface hydrophobicity of sphingomonads
Source: mSystems. 2023 Nov 1;8(6):e00862-23. doi: 10.1128/msystems.00862-23 (PMC10734547; doi:10.1128/msystems.00862-23)
Supplement: Tables S1 and S2 — The enriched KEGG pathways and distributions of polysaccharide synthesis genes. [file msystems.00862-23-s0008.docx]

**TABLE S1** The enriched KEGG pathways in C1 and C2△*p3_rep* compared with C2

| Pathway | DEGs with pathway annotation | All genes with pathway annotation | P value | Q value |
| --- | --- | --- | --- | --- |
| **C1 VS C2** |  |  |  |  |
| Bacterial secretion system (ko03070) | 24 | 54 | 6.646E-26 | 2.592E-24 |
| **C2△*p3_rep* VS C2** |  |  |  |  |
| Bacterial secretion system (ko03070) | 17 | 54 | 0.00010 | 0.00891 |
| Polyketide sugar unit biosynthesis (ko00523) | 4 | 5 | 0.00095 | 0.04456 |
| Flagellar assembly (ko02040) | 10 | 33 | 0.00397 | 0.12348 |
| Cysteine and methionine metabolism (ko00270) | 9 | 29 | 0.00525 | 0.12348 |
| Folate biosynthesis (ko00790) | 8 | 28 | 0.01442 | 0.21628 |

**TABLE S2** The distributions of polysaccharide synthesis genes encoded by the plasmid p5 in the chromosomes and plasmids from sphingomonads.

| Gene ID | Annotation | Primary chromosome | Secondary chromosome | Plasmid |
| --- | --- | --- | --- | --- |
| P5_06 | *rfaG*; glycosyltransferase | 0 | 0 | 1 |
| P5_07 | *galE*, GALE; UDP-glucose 4-epimerase | 0 | 0 | 2 |
| P5_08 | group 1 glycosyl transferase | 0 | 0 | 2 |
| P5_10 | group 1 glycosyl transferase | 0 | 0 | 2 |
| P5_12 | *wza*, *gfcE*; polysaccharide biosynthesis/export protein | 9 | 0 | 3 |
| P5_13 | *rfbC*, *rmlC*; dTDP-4-dehydrorhamnose 3,5-epimerase | 27 | 0 | 6 |
| P5_14 | *rfbB*, *rmlB*, *rffG*; dTDP-glucose 4,6-dehydratase | 46 | 0 | 11 |
| P5_15 | *rfbD*, *rmlD*; dTDP-4-dehydrorhamnose reductase | 17 | 0 | 5 |
| P5_16 | *rfbA*, *rmlA*, *rffH*; glucose-1-phosphate thymidylyltransferase | 54 | 0 | 10 |
| P5_17 | *manB*; phosphomannomutase | 13 | 0 | 11 |
| P5_32 | *algA*, *xanB*, *rfbA*, *wbpW*, *pslB*; mannose-1-phosphate guanylyltransferase / mannose-6-phosphate isomerase | 13 | 0 | 3 |
| P5_38 | putative glycosyltransferase | 10 | 0 | 4 |
| P5_42 | *kpsE*; capsular polysaccharide transport system permease protein | 8 | 0 | 3 |
| P5_43 | *kpsM*; capsular polysaccharide transport system permease protein | 9 | 0 | 3 |
| P5_44 | *kpsT*; capsular polysaccharide transport system ATP-binding protein | 37 | 1 | 4 |
| P5_46 | family 2 glycosyl transferase | 0 | 0 | 1 |

The distributions were investigated by Blastp (identity ≥50%, coverage ≥50%) against the 462926 proteins encoded by 109 primary chromosomes, 18 secondary chromosomes, and 202 plasmids from sphingomonads (including strain C1).
